# Vitamin C promotes the proliferation and effector functions of human γδ T cells

**DOI:** 10.1038/s41423-019-0247-8

**Published:** 2019-06-06

**Authors:** Léonce Kouakanou, Yan Xu, Christian Peters, Junyi He, Yangzhe Wu, Zhinan Yin, Dieter Kabelitz

**Affiliations:** 1 Institute of Immunology, Christian-Albrechts-University Kiel and University Hospital Schleswig-Holstein Campus Kiel, Kiel, Germany; 2 The First Affiliated Hospital, Biomedical Translational Research Institute, Guangdong Province Key Laboratory of Molecular Immunology and Antibody Engineering, Jinan University, 510632 Guangzhou, China

**Keywords:** γδ T cells, Vitamin C, lymphocyte activation, adoptive T-cell transfer, Immunology, Lymphocytes, T cells, Gammadelta T cells

## Abstract

γδ T cells are of interest as effector cells for cellular immunotherapy due to their HLA-non-restricted lysis of many different tumor cell types. Potential applications include the adoptive transfer of in vitro-expanded γδ T cells. Therefore, it is important to optimize the culture conditions to enable maximal proliferative and functional activity. Vitamin C (L-ascorbic acid) is an essential vitamin with multiple effects on immune cells. It is a cofactor for several enzymes, has antioxidant activity, and is an epigenetic modifier. Here, we investigated the effects of vitamin C (VC) and its more stable derivative, L-ascorbic acid 2-phosphate (pVC), on the proliferation and effector function of human γδ T cells stimulated with zoledronate (ZOL) or synthetic phosphoantigens (pAgs). VC and pVC did not increase γδ T-cell expansion within ZOL- or pAg-stimulated PBMCs, but increased the proliferation of purified γδ T cells and 14-day-expanded γδ T-cell lines in response to γδ T-cell-specific pAgs. VC reduced the apoptosis of γδ T cells during primary stimulation. While pVC did not prevent activation-induced death of pAg-restimulated γδ T cells, it enhanced the cell cycle progression and cellular expansion. Furthermore, VC and pVC enhanced cytokine production during primary activation, as well as upon pAg restimulation of 14-day-expanded γδ T cells. VC and pVC also increased the oxidative respiration and glycolysis of γδ T cells, but stimulus-dependent differences were observed. The modulatory activity of VC and pVC might help to increase the efficacy of γδ T-cell expansion for adoptive immunotherapy.

## Introduction

Vitamin C (L-ascorbic acid) is an essential vitamin that must be obtained through appropriate nutrition or dietary sources. Vitamin C (VC) plays important roles in many different biological processes, spanning from stem cell differentiation to cancer cell biology.^1,2^ VC is a potent antioxidant and free radical scavenger, as well as an essential cofactor in many enzymatic reactions. Moreover, VC has recently been found to selectively kill colorectal cancer cells with KRAS or BRAF mutations.^3^ In addition, VC is an epigenetic modifier that regulates gene expression via DNA hydroxymethylation through modulation of ten-eleven translocation (Tet) enzymes.^4^ A few studies have investigated the effects of VC on lymphocyte activation and differentiation. VC has been found to promote the development of T cells from murine^5^ and human hematopoietic progenitor cells.^6^ VC at high concentrations reduces the viability of lymphocytes and inhibits lymphocyte proliferation in vitro,^7–9^ while optimal concentrations of VC promote B-cell proliferation in response to LPS^8^ and T-cell proliferation in response to the mitogen concanavalin A.^10^ VC also augments the in vitro proliferative capacity of human NK cells.^11^ Furthermore, it has been reported that VC increases the stability and function of regulatory T cells through Tet-dependent demethylation of the *FoxP3* regulatory elements.^12,13^ Overall, it is clear that VC can modulate multiple lymphocyte functions and therefore might exert beneficial effects in the context of cellular immunotherapy.^14^

γδ T cells have recently attracted substantial interest as potential effector cells in cancer immunotherapy, mainly because of their potent cytotoxicity toward tumor cells and their MHC/HLA-independent mode of action.^15^ The dominant subset of γδ T cells in human peripheral blood expresses a Vγ9Vδ2-encoded T-cell receptor (TCR), while Vδ1 γδ T cells are more abundant in (mucosal) tissues.^15^ Both subsets can efficiently kill tumor cells and may play a role in antitumor immunity.^16^ Vγ9Vδ2 γδ T cells recognize microbial pyrophosphate molecules (“phosphoantigens”, pAgs), which are intermediates of the prokaryotic non-mevalonate pathway of isoprenoid biosynthesis secreted by many bacteria and some parasites. The most potent microbial pAg is (*E*)-4-hydroxy-3-methyl-but-2-enyl pyrophosphate (HMBPP).^17^ The synthetic bromohydrin pyrophosphate (BrHPP) is less potent than HMBPP but also selectively activates Vγ9Vδ2T cells.^18^ Eukaryotic cells produce homologous pyrophosphates in the mevalonate pathway of cholesterol synthesis (isopentenyl pyrophosphate, IPP), which, however, require much higher (micromolar) concentrations for activation of γδ T cells.^19^ Importantly, the mevalonate pathway is frequently dysregulated in tumor cells, resulting in overproduction of endogenous IPP, which can then activate Vγ9Vδ2 T cells.^20^ While the recognition of pAg does not involve HLA molecules, an indispensable role for members of the butyrophilin protein family (specifically for the BTN3A isoforms) has been identified. Accumulating evidence indicates that binding of pyrophosphates to the intracellular domain of BTN3A1 induces a conformational change in the extracellular domain, which is then recognized by the Vγ9Vδ2 TCR.^21^ The endogenous levels of pyrophosphates can be manipulated by pharmacological approaches, specifically by nitrogen-containing aminobisphosphonates such as zoledronate (zoledronic acid, ZOL), which are in clinical use for the treatment of bone diseases. ZOL inhibits farnesyl pyrophosphate synthase in the mevalonate pathway, resulting in the upstream accumulation of IPP and increased susceptibility of tumor cells to γδ T-cell-mediated lysis.^22^ Based on this concept, ZOL has been used for the in vivo activation of γδ T cells in cancer patients, as well as for the in vitro expansion of Vγ9Vδ2 T cells for subsequent adoptive transfer. Clinical responses have been observed in some patients with both strategies in several small-scale studies across different tumor entities.^23^ However, the efficacy of γδ T-cell-based immunotherapy must be improved to reach durable clinical responses. Therefore, it is important to consider that, in some instances, intratumoral γδ T cells might also promote tumor development and metastasis by multiple mechanisms, including local IL-17 production.^15,24^

In the present study, we report that VC and its more stable and less toxic derivative L-ascorbic acid 2-phosphate (pVC) enhance the activation and differentiation of human Vγ9Vδ2 γδ T cells under different in vitro culture conditions, i.e., during primary stimulation as well as upon restimulation of short-term-expanded γδ T-cell lines. Our results suggest novel strategies to optimize the generation of effective Vγ9Vδ2 T cells for adoptive transfer into cancer patients.

## Materials and methods

### Cell purification and cell culture

The use of blood from healthy adult blood donors was approved by the Institutional Review Boards of the Medical Faculty of the University of Kiel (D546/16) and of Jinan University, Guangzhou. Peripheral blood mononuclear cells (PBMCs) were isolated from leukocyte concentrates (provided by the Institute of Transfusion Medicine, UKSH, Kiel) or from the heparinized blood of healthy donors by Ficoll-Hypaque (Biochrom, Berlin, Germany) density gradient centrifugation. Total γδ T cells were positively isolated by magnetic cell sorting following the manufacturer’s instructions (Miltenyi Biotec, Bergisch-Gladbach, Germany). PBMCs were stimulated at the indicated final concentrations with pAg HMBPP (Echelon Biosciences, Salt Lake City, USA), BrHPP (kind gift of Innate Pharma, Marseille, France) or with ZOL (Novartis, Basel, Switzerland) and 50 IU/mL recombinant human IL-2 (Novartis) in 96-well round-bottom microculture plates. PBMCs (1 × 10^6^ cells/mL) and purified γδ T cells (1 × 10^6^ cells/mL) were cultured in RPMI 1640 medium supplemented with 2 mM L-glutamine, antibiotics, 10 mM HEPES and 10% heat-inactivated fetal bovine serum and incubated at 37 °C in a humidified atmosphere of 5% CO_2_ in air. In some experiments, PBMCs were cultured at 3–4 × 10^6^ cells/mL in 24-well plates and stimulated with ZOL (50 μM; Merck, Darmstadt, Germany) in the presence of 100 IU/mL IL-2. Short-term (12–14 days) lines of Vγ9Vδ2T cells were established by stimulating PBMCs with 2.5 µM ZOL (Novartis) in the presence of 50 IU/mL IL-2. IL-2 was added every 2–3 days over a culture period of 14 days. Lines were used for experiments when γδ T cells represented >90% of the total cell population, as determined by the proportion of Vγ9 or Vδ2 T cells. The short-term-expanded γδ T-cell lines (4 × 10^4^/well) were restimulated in 96-well round-bottom plates with 300 nM BrHPP in the presence of 50 IU/mL IL-2. Vitamin C (A4544) and phospho-modified vitamin C (A8960; both from Merck) were dissolved in water and added at the concentrations indicated in the Results section.

### Flow cytometry

Cells were stained in different combinations with fluorochrome-conjugated monoclonal antibodies directed against CD3 (clones SK7 and UCHT1), CD80 (clone L307.4), CD45RA (clone L48), CD27 (clone M-T271), CD69 (clone L78), GATA-3 (clone L50-823), and TNF-α (clone MAb11), all from BD Biosciences (Heidelberg, Germany); T-bet (clone 4B10), IFN-γ (clone B27), CCR7 (clone G043H7), TCR Vδ2 (clone B6), and Ki-67 (clone Ki-67) from Biolegend (London, UK); CD86 (clone 2331[FUN-1]) from R&D Systems (Wiesbaden, Germany); TCR Vγ9 [clone 7A5^25^] and their corresponding isotype controls (from BD Biosciences or Biolegend). For the detection of transcription factors, cells were fixed and permeabilized using Foxp3 transcription factor staining buffer (eBioscience; Thermo Fisher, Waltham, USA) according to the manufacturer´s instructions. For the intracellular detection of Ki-67 antigen, cells were subjected to ethanol fixation. For the detection of intracellular cytokines, Vγ9Vδ2 T cells expanded from PBMCs for 14 days with ZOL were stimulated for 6 h with 5 μg/mL plate-bound anti-human CD3 (eBioscience, clone OKT3) and 1 μg/mL soluble anti-human CD28 (eBioscience, clone CD28.2) antibodies in the presence of 5 μg/mL brefeldin A. Thereafter, cells were fixed and permeabilized after cell surface staining using a Lysis Solution (BD Biosciences) and Permeabilization Solution 2 (BD Biosciences). Cells were acquired on an LSRII Fortessa (BD Biosciences) and data were analyzed with FlowJo v. 10 (FlowJo, LLC). Apoptotic cell death was quantified using a propidium iodide (PI)/annexin V-FITC staining kit (MabTag, Friesoythe, Germany) according to the manufacturer´s protocol. We analyzed Vγ9Vδ2 T cells at days 14 and 21 after primary stimulation of PBMCs with ZOL. We also analyzed activation-induced cell death in short-term-expanded γδ T cell lines.^22^ To this end, γδ T cells expanded for 14 days were preincubated (or not) for 20 h with pVC before restimulation with BrHPP (or medium). After 20 h, cells were harvested and washed with 1× binding buffer containing 10 mM HEPES (pH 7.4), 140 mM NaCl, and 5 mM CaCl_2_. Thereafter, cells were resuspended in 1× binding buffer and stained with 100 µL of annexin V-FITC (1:50) and 1 µg/mL PI for 15 min at room temperature in the dark and immediately subjected to flow cytometry analysis. The level of intracellular ROS was measured using a ROS detection reagent (Thermo Fisher) according to the manufacturer’s guidelines. ROS levels were measured with an LSRII Fortessa and data were analyzed with FlowJo v. 10. For the determination of γδ T-cell expansion, a previously described flow cytometry-based method, termed standard cell dilution assay, was used to measure the absolute number of proliferating Vγ9 T cells per microculture well.^26^ Briefly, expanded cells were harvested at the indicated time points, washed, and stained with AF488-labeled anti-Vγ9 (7A5) and PE-conjugated anti-CD3 mAbs for 30 min. Shortly before analysis, PI (0.2 µg/mL) and a known number of APC-labeled and fixed standard cells were added. The absolute cell number of viable Vγ9 T cells was determined based on the ratio of AF488^+^ Vγ9 cells/APC^+^ standard cells. In some experiments, the absolute number of viable γδ T cells was counted microscopically after eosin dye exclusion of dead cells.

### Cell cycle analysis

Vγ9Vδ2 T cell lines were stimulated at 1 × 10^6^ cells/mL with BrHPP (300 nM) for 3 days after a 20-h  pretreatment with pVC. Cells were washed twice and resuspended in 0.5 mL of cold 5 mM EDTA-containing PBS. Cells were fixed by adding 0.5 mL of ethanol (100%). After 30 min of incubation at room temperature, the cells were washed and resuspended in 5 mM EDTA-containing PBS. Cells were incubated with 1 mg/mL RNase A (Qiagen, Hilden, Germany) and 50 µg/mL PI for 1 h at room temperature. DNA distribution was analyzed with a FACSCalibur flow cytometer (BD Biosciences).

### Mitochondrial imaging

Fourteen-day-expanded Vγ9Vδ2 T cells were stained with MitoTracker™ Red and DAPI (Thermo Fisher). The number and distribution of mitochondria wereobserved by confocal microscopy, and the fluorescence intensity of mitochondria was analyzed by Leica LAS AF Lite software.

### Metabolic activity

The oxygen consumption rate (OCR) and extracellular acidification rate (ECAR) were measured in XF base medium (Seahorse Bioscience; Agilent, Santa Clara, USA) containing 10 mM glucose, 2 mM L-glutamine, and 1 mM sodium pyruvate (all from Merck), under basal conditions and in response to 1 μM oligomycin (Seahorse Bioscience) to block ATP synthesis, 0.25 μM FCCP (Seahorse Bioscience) to uncouple ATP synthesis from the electron transport chain, and 0.5 μM rotenone and antimycin A (Seahorse Bioscience) to block complex I and III of the electron transport chain, on an XF-96 Extracellular Flux Analyzer (Seahorse Bioscience) according to the manufacturer’s recommendations.

### Measurement of cytokine production

γδ T cells (5 × 10^4^/well) freshly isolated from PBMCs by magnetic sorting were stimulated with BrHPP in IL-2-containing medium in the presence or absence of pVC. On day 8, the supernatants were collected and assessed for cytokine content with a LEGENDplex^TM^ human Th cytokine panel (BioLegend). Based on these results, levels of IFN-γ and IL-13 were additionally quantified by ELISA (Duoset; R&D Systems, Wiesbaden, Germany).

### Statistical analysis

Statistical significance was calculated with the paired, two-tailed Student’s *t*-test using Microsoft Excel 2007 and ANOVA when comparing more than two groups using GraphPad Prism version 6.01. Values of *p* < 0.05 were considered indicative of significance and are displayed as * for *p* < 0.05, ** for *p* < 0.01, and *** for *p* < 0.001. The results in graphs represent the mean/median ± SD/SEM, with the numbers of independent experiments and experimental replicates indicated in each figure legend.

## Results

### Differential effects of VC and phospho-Vitamin C (pVC) on γδ T-cell expansion

**Fig. 1:**
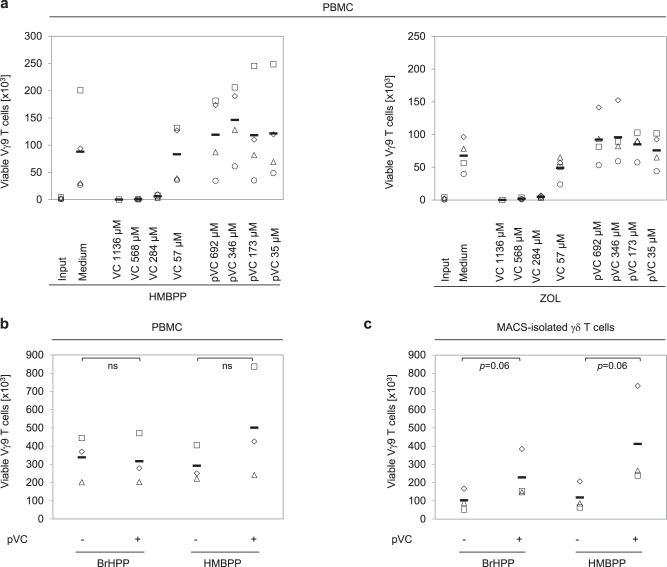
Distinct effects of VC and pVC on the in vitro expansion of Vγ9Vδ2 T cells. **a** PBMCs obtained from four healthy donors were stimulated with HMBPP (left panel) or ZOL (right panel) in the presence of IL-2. VC or pVC was added at the indicated concentrations. The number of viable Vγ9 T cells per microculture well was determined in triplicate by flow cytometry after 7 days of culture. Each symbol indicates an individual healthy donor. Horizontal bars represent the mean values. **b** PBMCs from three healthy donors were stimulated with BrHPP or HMBPP and IL-2 in the absence or presence of 50 μg/mL (173 μM) pVC. The number of viable Vγ9 T cells per microculture well was determined in triplicate by flow cytometry after 7 days of culture. Each symbol indicates an individual healthy donor. Horizontal bars represent the mean values. **c** Purified γδ T cells (50,000/well) from the same donors as in (**b**) were stimulated with BrHPP or HMBPP and IL-2 in the absence or presence of 50 μg/mL (173 μM) pVC. The number of viable Vγ9 T cells per microculture well was determined in triplicate by flow cytometry after 7 days of culture. Each symbol indicates an individual healthy donor. Horizontal bars represent the mean values. Statistical significance was calculated with the paired, two-tailed Student’s *t*-test. ns, not significant

**Fig. 2:**
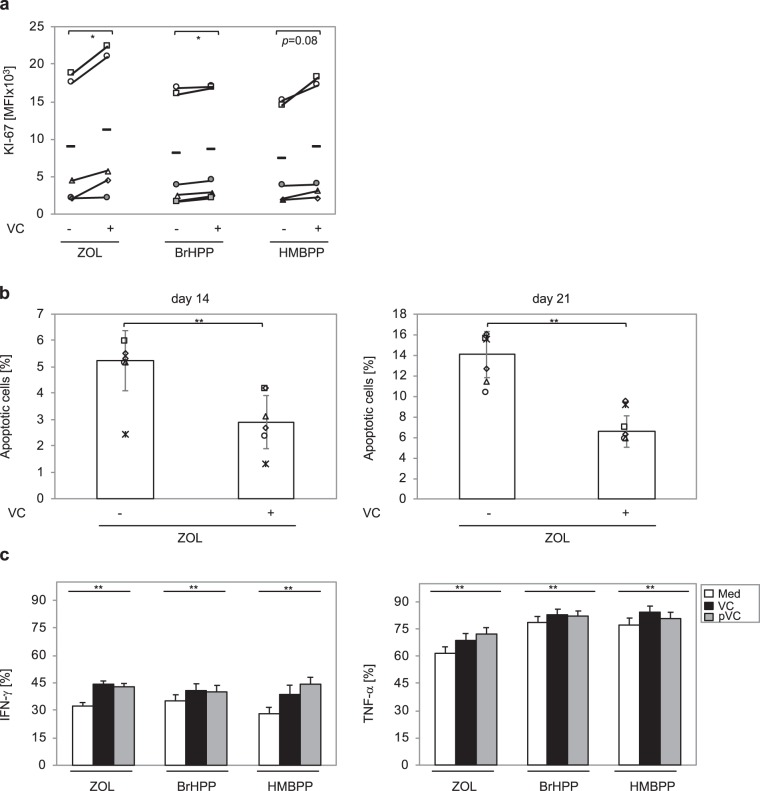
Effect of VC and pVC on Ki-67 expression, cell death and cytokine expression. **a** PBMCs from healthy donors (ZOL and HMBPP: *n* = 5; BrHPP: *n* = 6) were stimulated with the indicated stimuli and IL-2 in the absence or presence of 12.5 μg/mL (70 μM) VC. After 14 days, intracellular Ki-67 expression was determined by flow cytometry on Vδ2-gated γδ T cells. The results are shown as the mean fluorescence intensity (MFI) of Ki-67 expression. **b** Analysis of apoptotic γδ T cells (annexin V^+^/PI^+^) after 14 (left) and 21 (right) days of ZOL stimulation of PBMCs (*n* = 6). **c** Intracellular expression of IFN-γ (left) and TNF-α (right) was measured after 14 days following stimulation for 6 h with anti-CD3/CD28 antibodies. The results are displayed as the proportion of positive cells (*n* = 9). **a**, **b** Statistical significance was calculated with the paired, two-tailed Student’s *t*-test. **c** Statistical significance between the groups was analyzed by ANOVA. *p < 0.05 and **p < 0.01

L-ascorbic acid (VC) is an established constituent of cell culture media used for stem cell differentiation. In these systems, the phospho-modified derivative, L-ascorbic acid 2-phosphate (pVC), is generally used because it is more stable and less toxic than VC at higher concentrations.^27^ In the first set of experiments, we compared the effects of VC and pVC over wide concentration ranges on the selective activation and short-term expansion of Vγ9Vδ2 T cells within PBMCs. To this end, PBMCs from healthy donors containing 2–4% γδ T cells were stimulated with optimal concentrations (10 nM) of HMBPP or ZOL (2.5 μM) and IL-2 (50 IU/mL). In cultures of PBMCs, ZOL activates Vγ9Vδ2 T cells due to the accumulation of IPP in monocytes.^28^ The selective expansion of γδ T cells was determined by flow cytometry by measuring the absolute number of viable Vγ9 T cells after 7 days of culture. As shown in Fig. 1a, Vγ9 T cells within PBMCs strongly expanded in response to both HMBPP (left panel) and ZOL (right panel). The overall proliferation was not significantly influenced by pVC over a concentration range of 35–692 μM. In contrast, VC (tested over a concentration range of 57–1136 μM) inhibited γδ T-cell expansion in response to both stimuli at higher concentrations. Based on these results, we used pVC at 173 μM (50 μg/mL) and VC at 70 μM (12.5 μg/mL) in all subsequent experiments. Next, we compared the effect of pVC on the proliferation of purified γδ T cells in response to the pAgs HMBPP and BrHPP. While low numbers of purified γδ T cells require the presence of accessory cells for activation by HMBPP,^29^ the high γδ T cell numbers per microculture well used here (50,000 purified γδ T cells per well) enabled pAg-induced γδ T-cell proliferation in the absence of accessory cells. As shown in Fig. 1b (and consistent with Fig. 1a), pVC did not significantly modulate γδ T-cell expansion within PBMCs stimulated with BrHPP or HMBPP. However, the proliferation of purified γδ T cells in response to both stimuli was increased by pVC, even though this increase did not reach statistical significance (Fig. 1c). We measured the absolute cell numbers by a different method, i.e., microscopy-based cell counting after eosin dye exclusion, and found the expansion of purified γδ T cells in response to BrHPP was significantly increased (Supplementary Fig. S1). Despite the lack of a significant growth-promoting effect of VC on γδ T-cell expansion within ZOL-stimulated PBMCs at day 7 (Fig. 1a), VC increased the intensity of Ki-67 expression measured at later time points (day 12) on γδ T cells within PBMCs stimulated with ZOL and pAgs (HMBPP and BrHPP) (Fig. 2a). Taken together, these results indicate that VC has a narrow concentration window for in vitro use, while the phospho-modified derivative pVC can be applied over a wider concentration range with no toxic effects. Both preparations have only limited effects on the proliferative expansion of γδ T cells during primary in vitro activation by ZOL or pAgs. A direct growth-promoting effect of pVC on pAg-activated purified γδ T cells (Fig. 1c and Supplementary Fig. S1) seems to be abrogated in the presence of monocytes, such as those present in PBMCs (Fig. 1a, b).

### Effect of VC and pVC during γδ T-cell expansion within ZOL- and pAg-stimulated PBMCs

**Fig. 3:**
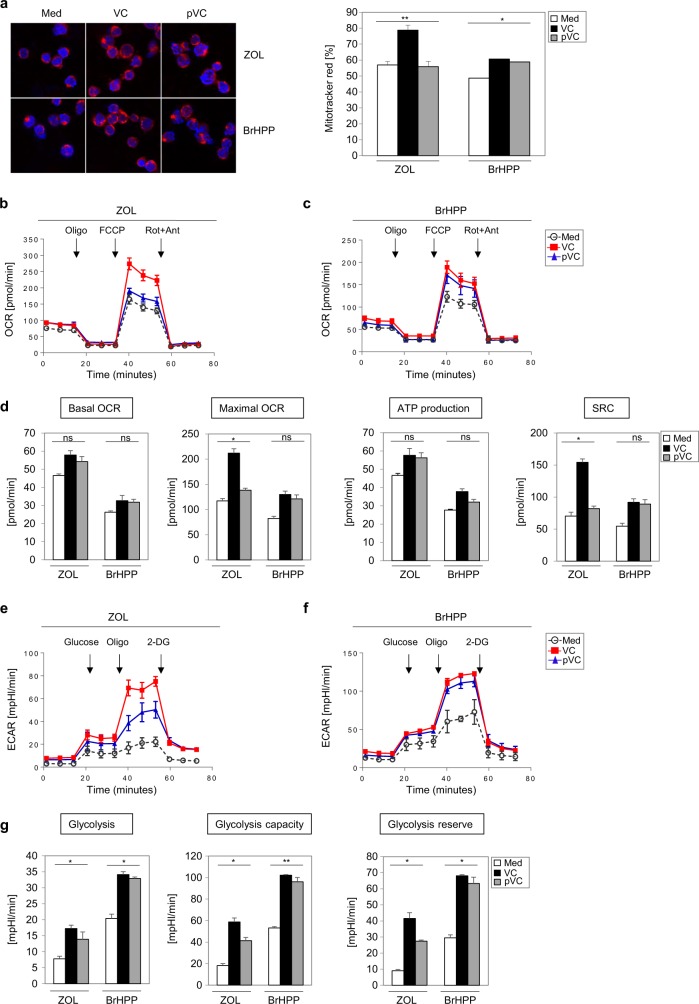
Differential effect of VC and pVC on the metabolic activity of γδ T cells during primary activation. PBMCs from healthy donors were activated for 14 days with ZOL or BrHPP in the presence of IL-2 and the additional presence or absence of 12.5 μg/mL (70 μM) VC or 50 μg/mL (173 μM) pVC, as indicated. **a** γδ T cells were stained with TCR Vδ2-PE, MitoTracker^TM^ Red FM, and DAPI. The distribution of mitochondria was observed by confocal microscopy (left), and the fluorescence intensity of mitochondria was measured by flow cytometry (right; mean ± SEM of five to eight experiments). **b**–**d** Oxygen consumption rate (OCR), ATP production, spare respiratory capacity (SRC), and **e**–**g** glycolysis were measured after 14 days using an XF-96 Extracellular Flux Analyzer (*n* = 3). Error bars represent SEM. Statistical significance between groups was calculated by ANOVA. *p < 0.05 and **p < 0.01

PBMCs were activated with ZOL in IL-2-containing medium in the presence or absence of VC or pVC. Culture medium containing fresh IL-2 and VC or pVC was exchanged every 3 days. At these later time points, γδ T cells expanded with VC displayed reduced numbers of annexin V^+^/propidium iodide^+^ (PI^+^) apoptotic cells at day 14 but also at day 21 (Fig. 2b). After 14 days, expanded γδ T cells were restimulated for 6 h with anti-CD3/CD28 antibodies and brefeldin A before intracellular staining for cytokines was performed. The gating strategy for intracellular cytokine analysis is shown in Supplementary Fig. S2. As illustrated in Fig. 2c, γδ T cells activated within PBMCs by ZOL, BrHPP, or HMBPP contained increased proportions of IFN-γ and TNF-α-producing cells. Next, we analyzed the effects of VC and pVC on the mitochondrial mass, oxidative respiration and glycolysis of γδ T cells within PBMCs stimulated for 12 days with ZOL, BrHPP, or HMBPP. The results for ZOL and BrHPP are shown in Fig. 3, and the results for HMBPP are shown in Supplementary Fig. S3. As presented in Fig. 3a, VC but not pVC increased mitochondrial mass upon ZOL stimulation as revealed by MitoTracker^TM^ Red FM and DAPI staining. In this respect, both VC and pVC had a modest effect upon BrHPP stimulation. Furthermore, γδ T cells expanded with ZOL in the presence of VC also exhibited increased oxidative respiration, as revealed by an increased OCR and spare respiratory capacity (Fig. 3b, d), and increased glycolysis, as revealed by increased ECAR (Fig. 3e, g). In comparison to ZOL, BrHPP induced less oxidative respiration (Fig. 3c, d) but increased glycolysis (Fig. 3f, g), both of which were similarly modulated by VC and pVC (i.e., a moderate increase in oxidative respiration but a strong increase in glycolysis) (Fig. 3d, g). Comparable results were obtained when HMBPP was used instead of BrHPP (Supplementary Fig. S3). Taken together, these data show that VC promotes metabolic activity associated with reduced cell death and increased cytokine production during primary stimulation of human γδ T cells.

### pVC promotes the expansion of short-term-expanded Vγ9Vδ2 T- cell lines upon restimulation

**Fig. 4:**
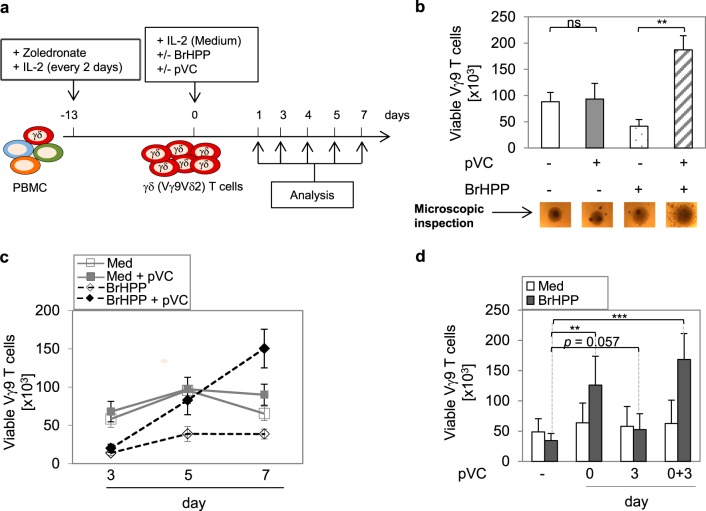
pVC promotes the proliferation of BrHPP-restimulated γδ T-cell lines. **a** Experimental conditions: PBMCs from healthy donors were stimulated for 12–13 days with 2.5 μM ZOL and 50 IU/mL IL-2 to generate short-term Vγ9Vδ2 T cell lines. Subsequently, the generated γδ T cells (4 × 10^4^/well) were left unstimulated or were restimulated for 7 days with BrHPP in the presence (or not) of 50 μg/mL (173 μM) pVC. Thereafter, the absolute number of viable Vγ9 T cells was determined by flow cytometry. **b** Summary bar graph of the number (mean ± SD) of viable Vγ9 T cells from six independent experiments. Lower part: microscopic inspection of γδ T-cell proliferation at 50× magnification. **c** Kinetics of Vγ9 T cell expansion, represented by mean values ± SD of viable cell numbers from three independent experiments. **d** Time window for the effect of pVC (*n* = 6 with mean ± SD). All samples were assessed in triplicate. Med, IL-2-containing medium. Statistical significance was calculated with the paired, two-tailed Student’s *t*-test. *p < 0.05, **p < 0.01, and ***p < 0.001, ns: not significant

In the following series of experiments, we used only the less toxic and more stable pVC, which has been reported to promote DNA synthesis and mammalian cell differentiation.^27,30^ Here, we tested the effect of pVC on the proliferation of short-term-expanded Vγ9Vδ2 T cell lines following restimulation with BrHPP for 7 days. To this end, γδ T-cell lines were established by culturing PBMCs with ZOL and repeatedly adding IL-2 for 12–13 days in the absence of pVC (see experimental scheme in Fig. 4a). The purity of these γδ T-cell lines, as determined by the proportion of Vγ9 or Vδ2 T cells, routinely exceeded 90%. These short-term γδ T-cell lines were washed and then recultured in IL-2-containing medium in the absence or presence of BrHPP and/or pVC. The absolute cell number of viable Vγ9 T cells was determined by flow cytometry. As expected, restimulation with BrHPP inhibited the proliferation of Vγ9 T cells due to the induction of activation-induced cell death (AICD),^25^ whereas the additional presence of pVC rescued and significantly increased the proliferative expansion of Vγ9 T cells after restimulation (Fig. 4b). Inspection of cell cultures by microscopy indicated that this effect was associated with increased cell aggregate formation when pVC was present together with BrHPP (Fig. 4b, lower part). In contrast, pVC had no effect on Vγ9 T cell expansion in the absence of BrHPP. To investigate the time point during which pVC contributed most to γδ T-cell proliferation, Vγ9Vδ2 T cell lines were left unstimulated or were restimulated with BrHPP in IL-2-containing medium in the absence or presence of pVC, and the expansion of Vγ9 T cells was determined at different time points. As shown in Fig. 4c, the enhancing effect of pVC was most obvious when cellular expansion (i.e., the number of viable Vγ9 T cells per microculture well) was analyzed at day 7 after restimulation. Next, we determined the time window during which pVC exerted its enhancing effect. To this end, short-term-expanded γδ T-cell lines were left unstimulated or were restimulated with BrHPP in IL-2-containing medium. pVC was added at different time points, i.e., day 0, day 3, or day 0 plus day 3. The proliferation of Vγ9 T cells was again quantified after 7 days. We observed that pVC was required at the initiation of the cell culture (day 0) to enhance γδ T-cell proliferation (Fig. 4d).

### pVC-enhanced γδ T-cell proliferation is not due to the inhibition of cell death

**Fig. 5:**
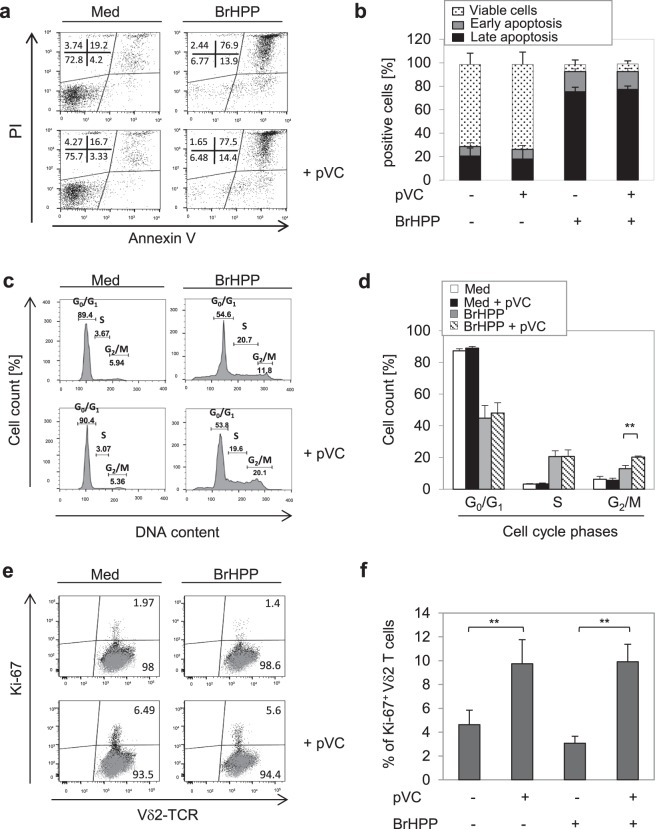
pVC promotes the proliferation of BrHPP-restimulated γδ T cells not by preventing apoptosis but by inducing cell cycle progression. **a** Short-term-expanded Vγ9Vδ2T cells were treated (where indicated) with pVC for 20 h followed by BrHPP stimulation for another 20 h. Thereafter, cells were stained with annexin V-FITC/PI and analyzed by flow cytometry. Left: dot plots representative of one out of four independent experiments are shown. Numbers in the indicated area in the FACS plots refer to the percentage of positive cells. **b** Summary bar graphs of four independent experiments (mean ± SD) showing the frequency of viable cells (annexin V^-^/PI^-^), early apoptotic cells (annexin V^+^/PI^-^) and late apoptotic cells (annexin V^+^/PI^+^). **c** Vγ9Vδ2 T cells were left unstimulated or were restimulated with BrHPP for 3 days after a 20-h pretreatment with pVC. The cell cycle distribution of living cells obtained by gating on the forward/side scatter was determined using PI staining and flow cytometry. Histograms of one representative experiment of four independent experiments are shown. **d** Bar graphs summarize the percentage (mean ± SD) of cells in each phase of the cell cycle; *n* = 4. **e** Representative dot blots of Ki-67 expression in viable Vδ2 T cells (determined by FACS gating) at day 7 after restimulation with BrHPP in the absence or presence of pVC. **f** Percentages (mean ± SEM) of Ki-67^+^ Vδ2 T cells from five independent experiments. Statistical significance was calculated with the paired, two-tailed Student’s *t*-test. **p < 0.01

TCR-dependent restimulation of activated γδ T cells is known to induce AICD.^25^ To assess whether pVC attenuated the cell death of BrHPP-restimulated Vδ2 T cells, we analyzed cell death induction by flow cytometry using combined annexin V and PI staining.^31^ Cells in the early apoptotic phase are annexin V^+^/PI^−^, those in the necroptotic phase are annexin V^-^/PI^+^, while dead cells (late apoptosis and necroptosis) stain positively for both markers.^31,32^ Vγ9Vδ2 T cell lines, pretreated (or not) with pVC, were left unstimulated or were restimulated with BrHPP in IL-2-containing medium. Cell death analysis was performed after 20 h. Dot blots of one representative experiment are presented in Fig. 5a, and a summary of four experiments is presented in Fig. 5b. As expected, BrHPP induced significant cell death (~77% PI^+^/annexin V^+^ cells), which, however, was not inhibited by pVC. Moreover, we also did not observe any effect of pVC on the appearance of early apoptotic (PI^-^/annexin V^+^) or necrotic cells (PI^+^/annexin V^-^). Since TCR stimulation also induces the production of reactive oxygen species (ROS), and VC is a well-known antioxidant, we also analyzed intracellular ROS production by flow cytometry. Consistent with the antioxidant activity of VC, we found that spontaneous and BrHPP-induced ROS production were significantly reduced by pVC (Supplementary Fig. S4). However, the antioxidant activity did not prevent BrHPP-induced cell death, as analyzed by annexin V/PI staining. Together, these results suggest that pVC rescues Vγ9Vδ2 T cells and increases their proliferation upon TCR restimulation by a mechanism independent of inhibition of apoptosis. Therefore, we explored further mechanisms underlying the pVC-increased proliferation.

### pVC promotes the cell cycle progression of restimulated γδ T cells

To further investigate the mechanism underlying the pVC-mediated enhanced proliferation of BrHPP-restimulated γδ T cells, we performed a cell cycle analysis by flow cytometry. To this end, Vγ9Vδ2 T cell lines were pretreated (or not) for 20 h with pVC before restimulation with BrHPP or medium only. Cell cycle analysis was performed after 3 days. Histograms of a representative experiment are depicted in Fig. 5c and a summary of four experiments is shown in Fig. 5d. In the presence of pVC, a significant shift toward an increased proportion of cells in the G_2_/M phase was observed in the BrHPP-restimulated cell cultures, as determined 3 days after restimulation. In addition to the cell cycle analysis on day 3, we also analyzed the expression of the cell proliferation marker Ki-67 on day 7. Ki-67 is expressed by cycling cells, i.e., those in S, G_2_, and M phases, but is absent in the G_0_ phase.^33,34^ In the presence of pVC, an increased proportion of Ki-67^+^ Vδ2 T cells expressed Ki-67, irrespective of whether they were restimulated with BrHPP or not (Fig. 5e, f). Taken together, these results suggest that pVC promotes the proliferation of restimulated Vγ9Vδ2 T cells not by preventing apoptosis but rather by inducing cell cycle progression.

### Modulation of surface markers and functions of Vγ9Vδ2 T cells in the presence of pVC

**Fig. 6:**
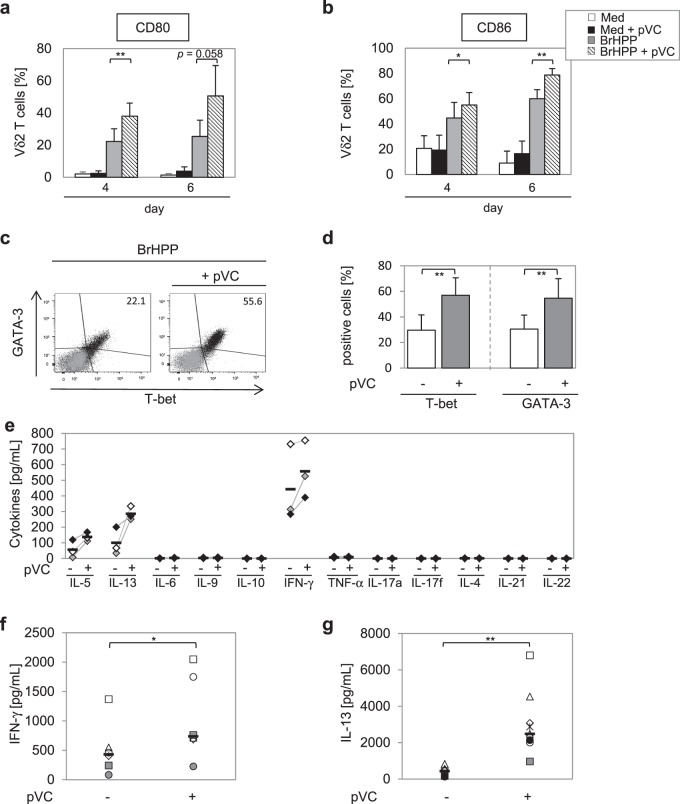
Modulation of surface markers, transcription factor expression, and cytokine secretion by pVC. **a**, **b** Twelve-day-expanded γδ T-cell lines were restimulated with BrHPP in the presence or absence of 50 μg/mL (173 μM) pVC. Four and six days after restimulation, cell surface expression of **a** CD80 (clone L307.4) and **b** CD86 (clone 2331[FUN-1]) was measured by flow cytometry. Summary bar graphs of four independent experiments (mean ± SD) showing the frequency of positive cells among gated Vδ2 T cells. **c**, **d** γδ T cells freshly purified from PBMCs were stimulated with BrHPP in IL-2-containing medium in the presence or absence of pVC. At day 8, cells were harvested, stained intracellularly for GATA-3 (clone L50-823) and T-bet (clone 4B10) (black) and their corresponding isotype controls (gray). **c** Representative dot plots of one out of six independent experiments are shown. **d** Summary bar graphs represent the mean values ± SD of six independent experiments. **e** The indicated cytokines present in the supernatants of the cell cultures in (**c**, **d**) were measured by LEGENDplex^TM^ bead-based array assay (*n* = 3). **f**, **g** Quantification of IFN-γ (*n* = 6) and IL-13 (*n* = 9) by ELISA in the supernatants obtained on day 8 after initial stimulation. Statistical significance was calculated with the paired, two-tailed Student’s *t*-test. *p *<* 0.05 and **p *<* 0.01

Next, we investigated the influence of pVC on certain surface markers of short-term-expanded Vγ9Vδ2 T cell lines upon restimulation with BrHPP. The expression of the activation-related molecule CD69, costimulatory receptor ligands CD80 and CD86, memory markers CD45RA and CD27, and chemokine receptor CCR7 was assessed by flow cytometry on day 4 and day 6 after restimulation. The expression of CD69, CD45RA/CD27, and CCR7 was not modulated by pVC treatment (results for CD45RA/CD27 are shown in Supplementary Fig. S5b). Interestingly, however, the expression of CD80 and CD86 was significantly higher on BrHPP-restimulated Vδ2 T cells treated with pVC than on untreated cells. In the absence of BrHPP restimulation, pVC did not enhance the expression of CD80 or CD86. Dot blots of a representative FACS analysis are shown in Supplementary Fig. S5c and a summary of four experiments is shown in Fig. 6a (CD80) and 6b (CD86).

Finally, we analyzed the effect of pVC on the expression of lineage (Th1, Th2)-specifying transcription factors by flow cytometry and on cytokine secretion in cell culture supernatants using a bead-based immunoassay and an ELISA. To this end, freshly isolated γδ T cells were activated for 7 days with BrHPP in IL-2-containing medium in the presence or absence of pVC. As illustrated in a representative dot blot in Fig. 6c, both T-bet and GATA-3 were expressed in IL-2-expanded γδ T cells upon BrHPP stimulation. Interestingly, the addition of pVC led to a significant increase in Vδ2 T cells coexpressing GATA-3 and T-bet. A summary of six experiments is shown in Fig. 6d. Culture supernatants collected at the same time were analyzed for secreted cytokines using a bead-based cytokine immunoassay. The most abundant cytokines secreted were IFN-γ, IL-5, and IL-13 (Fig. 6e). Quantification of the key Th1 and Th2 cytokines IFNγ and IL-13 by ELISA confirmed that pVC significantly increased the levels of IFN-γ and particularly of IL-13 (Fig. 6e, f).

## Discussion

Several studies have demonstrated that L-ascorbic acid/VC, in addition to its role in cancer treatment,^35–38^ promotes mammalian cell differentiation and DNA synthesis.^6,27,30,32^ However, the impact of VC on the differentiation and effector function of human γδ T cells has not been addressed to date. Results from human hematopoietic stem cell differentiation as well as human corneal endothelial cell culture studies have demonstrated that L-ascorbic acid, if present in the less stable nonphosphorylated form (VC), has lower potency to induce cell proliferation than the phospho-modified derivative L-ascorbic acid 2-phosphate (pVC), which is also more stable in cell culture.^6,39^ In the present study, we used both forms of VC and analyzed the effects on γδ T-cell activation during primary activation of PBMCs or purified γδ T cells with γδ T cell selective stimuli (the pAgs HMBPP and BrHPP, and the aminobisphosphonate ZOL), as well as during restimulation of short-term-expanded γδ T-cell lines with BrHPP (i.e., under conditions where BrHPP triggers AICD).

We observed that at concentrations above 100 µM VC actually inhibited the proliferation of γδ T cells when PBMCs were stimulated with HMBPP or ZOL, in contrast to pVC, which showed no toxicity even at the highest tested concentration of 692 μM (Fig. 1). It has been shown that VC is quickly oxidized in culture medium, inducing toxic levels of ascorbyl radical and ROS products (mainly H_2_O_2_), which inhibit cell growth.^39^ In contrast, pVC is resistant to auto-oxidation in culture medium and is thereby thought to promote cell survival.^27,40^ Moreover, it has been reported that high levels of ROS inhibit glyceraldehyde-3-phosphatase dehydrogenase and thereby impede metabolic reprogramming.^41^ This notwithstanding, we found that a lower concentration of 70 μM VC increased cytokine induction, metabolic activity, and mitochondrial mass of γδ T cells expanding within ZOL-stimulated PBMCs and reduced their apoptotic death rate after 14 and 21 days of culture. The direct comparison of the effects of VC and pVC on the metabolic activity of ZOL- versus pAg-activated γδ T cells revealed remarkable differences. VC, but not pVC, increased the maximal OCR and mitochondrial spare respiration capacity of ZOL-activated γδ T cells. Interestingly, BrHPP (as well as HMBPP) induced higher glycolysis than ZOL, and glycolysis of BrHPP-activated γδ T cells was further increased by both VC and pVC treatment (Fig. 3). The reason for the differential stimulation of metabolic activity by ZOl versus BrHPP is not clear but might be related to the different modes of action of the two stimuli. Activation of γδ T cells by ZOL depends on metabolically active monocytes,^28^ which are not required for BrHPP stimulation. Further studies are also needed to investigate in more detail why VC and pVC have differential effects on some but not other parameters of metabolic activity. Nevertheless, our results clearly show that VC and its derivative can increase the mitochondrial respiratory capacity and glycolysis of γδ T cells generated from ZOL- or pAg-stimulated PBMCs during the primary activation period.

In addition to the studies on the modulation of γδ T-cell activation during primary in vitro activation, we also investigated the effect of pVC in a different setting where short-term-expanded γδ T-cell lines were restimulated with BrHPP in the absence or presence of pVC. We restricted this analysis to the use of the non-toxic pVC. In the presence of IL-2, preactivated γδ T cells rapidly undergo AICD upon TCR/CD3 engagement.^25,42^ Consistent with previous studies, we found that BrHPP restimulation induced apoptosis in nearly 80% of γδ T cells that rapidly progressed to loss of membrane integrity and cell death, as evidenced by annexin V/PI costaining. Interestingly, the addition of pVC drastically increased the number of proliferating γδ T cells within BrHPP-restimulated γδ T-cell cultures. Surprisingly, however, pVC treatment did not prevent or reduce the BrHPP-induced apoptosis of γδ T-cell cultures, which is in contrast to previous studies in other cell systems. As an example, pVC in combination with the antioxidant N-acetylcysteine has been reported to exert synergistic protection of human mesenchymal stem cells (hMSCs) against different forms of cell death.^32^ Similarly, VC has been found to enhance the in vitro proliferation of mouse αβ T cells by acting as a potent inhibitor of various forms of cell death.^43^ Our data with ZOL-stimulated PBMCs indicated that VC can attenuate γδ T-cell death during primary expansion (Fig. 2b). However, the AICD of short-term-expanded γδ T-cell lines upon BrHPP restimulation was not prevented by pVC, although we observed that pVC exerted antioxidant activity as it reduced spontaneous and BrHPP-induced ROS production (Supplementary Fig. S4). Rather than inhibiting cell death, we found that pVC enhanced the expansion of the BrHPP-restimulated γδ T cells by stimulating their cell cycle progression. The cell cycle includes four stages: G_1_, S, G_2_, and M.^44^ G_1_/S and G_2_/M phases are considered specific checkpoints that monitor the entire cell division.^45^ We observed a significant enrichment of Vγ9Vδ2 T cells restimulated with BrHPP in the G_2_/M phase in the presence of pVC. Consistently, we also observed that γδ T cells treated with pVC showed increased expression of Ki-67, which is a marker of cycling cells (S/G_2_/M).^33^ Interestingly, pVC alone increased Ki-67 expression (Fig. 5e, f), but not G_2_/M accumulation (Fig. 5c, d) of Vδ2T cells. This apparent discrepancy is most likely due to the different time points of analysis (cell cycle analysis was performed at day 3 after restimulation; Ki-67 expression analysis was performed at day 7). Taken together, our results indicate that pVC induces the expansion of restimulated Vγ9Vδ2 T cells not by inhibiting AICD but rather by promoting cell cycle progression. However, we cannot exclude the possibility that other factors, such as reduced ROS production upon BrHPP restimulation in the presence of pVC (Supplementary Fig. S4), might also play a role.

In addition to enhancing the expansion of Vγ9Vδ2 T cells following restimulation with BrHPP, we also observed that pVC upregulated the expression of costimulatory CD80 and CD86 (but not other analyzed molecules) on γδ T cells. We also noticed upregulated coexpression of the transcription factors T-bet and GATA-3 in the presence of pVC. γδ T cells display a high level of plasticity,^46^ which is reflected here by the simultaneous expression of Th1- and Th2-associated transcription factors. We also measured increased secretion of the key Th1/Th2 cytokines IFN-γ and IL-13 in the presence of pVC. While TNF-α was absent in the supernatant of BrHPP-restimulated short-term γδ T-cell lines (Fig. 6e), intracellular TNF-α expression was clearly detected during primary activation of γδ T cells in ZOL- or pAg-activated PBMCs (Fig. 2c). This apparent discrepancy is most likely related to the different experimental conditions. While γδ T cells among ZOL/pAg-activated PBMCs were restimulated with anti-CD3/CD28 mAb before intracellular staining for TNF-α, cell culture supernatants from BrHPP-restimulated short-term γδ T-cell lines were collected without prior anti-CD3/CD28 mAb activation. Taken together, our results demonstrate that both VC and pVC augment cytokine induction and secretion in human Vγ9Vδ2 T cells. The mechanistic basis for this finding is currently unclear but might involve epigenetic regulation since VC is known to regulate DNA hydroxymethylation via Tet enzymes.^4,13^ More specifically, Tet2 has been shown to regulate the activation of cytokine gene expression in T cells.^47^ However, VC can also regulate cytokine expression in a Tet-independent manner via the histone demethylase jumonji-C domain-containing protein 2 (jmjd2).^48^ Ongoing studies in our laboratories will address the impact of VC and pVC on genome-wide DNA methylation in γδ T cells.

In conclusion, our results demonstrate that VC and its derivative pVC significantly modulate the in vitro expansion and cytokine production of human Vγ9Vδ2 T cells. To apply adoptive Vγ9Vδ2 T cell transfer as an immunotherapeutic approach for cancer patients, large numbers of cells are required. Based on our current results, we suggest that the addition of VC or pVC to cell cultures for large-scale expansion requiring occasional restimulation will greatly improve the generation of sufficiently large numbers of Vγ9Vδ2 T cells. We consider it an additional benefit that the VC- and pVC-treated Vγ9Vδ2 T cells produce increased amounts of IFN-γ, which can clearly support antitumor immunity.^49^ It remains to be investigated if additional features of Vγ9Vδ2 T cells, such as their capacity for B cell help^50^ or antigen cross-presentation,^51^ are also modulated by VC. Finally, given that VC can also be easily administered in high concentrations in vivo,^35^ it might also be considered to support γδ T cell-based immunotherapy with the in vivo application of VC.

## Supplementary information

Sup Material
