# Supplementary material for: Vitamin C promotes the proliferation and effector functions of human γδ T cells
Source: Cell Mol Immunol. 2019 Jun 6;17(5):462–73. doi: 10.1038/s41423-019-0247-8 (PMC7192840; doi:10.1038/s41423-019-0247-8)
Supplement: Supplementary file 1 — Sup Material [file 41423_2019_247_MOESM1_ESM.pdf]

Vitamin C promotes the proliferation and effector functions of human γδ T cells

Léonce Kouakanou, Yan Xu, Christian Peters, Junyi He, Yangzhe Wu, Zhinan Yin, Dieter Kabelitz

Supplemental Material (Supplemental Figures S1 – S5)

Supplemental Figure S1

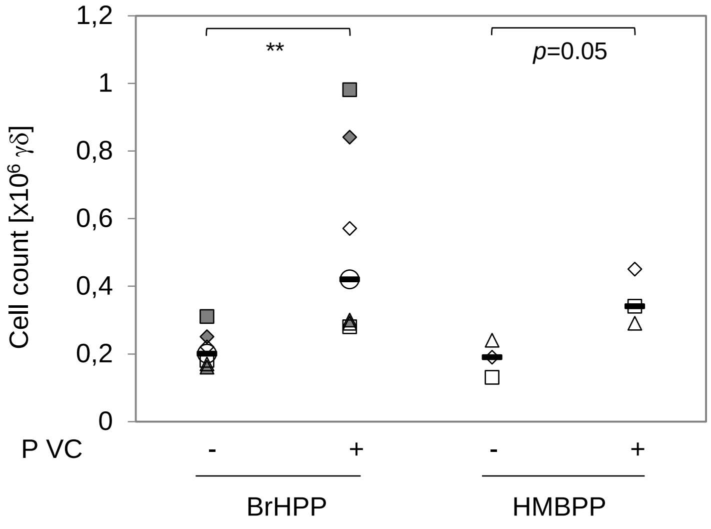

Supplementary Figure S1: pVC increases the *in vitro* growth of BrHPP-stimulated purified γδ T cells.

γδ T cells were purified from PBMC and activated with BrHPP or HMBPP in the presence of exogenous IL-2 and additional absence or presence of 50 µg/mL (173 µM) pVC (50.000 γδ T cells per well in 96-round bottom plates). After 7 d of culture, absolute cell counts were determined microscopically after exclusion of dead cells by eosin dye exclusion. BrHPP, n=7; HMBPP, n=3. Statistical significance was calculated with the paired, two-tailed Student's t-test. ** $p<0.01$

Supplemental Figure S2

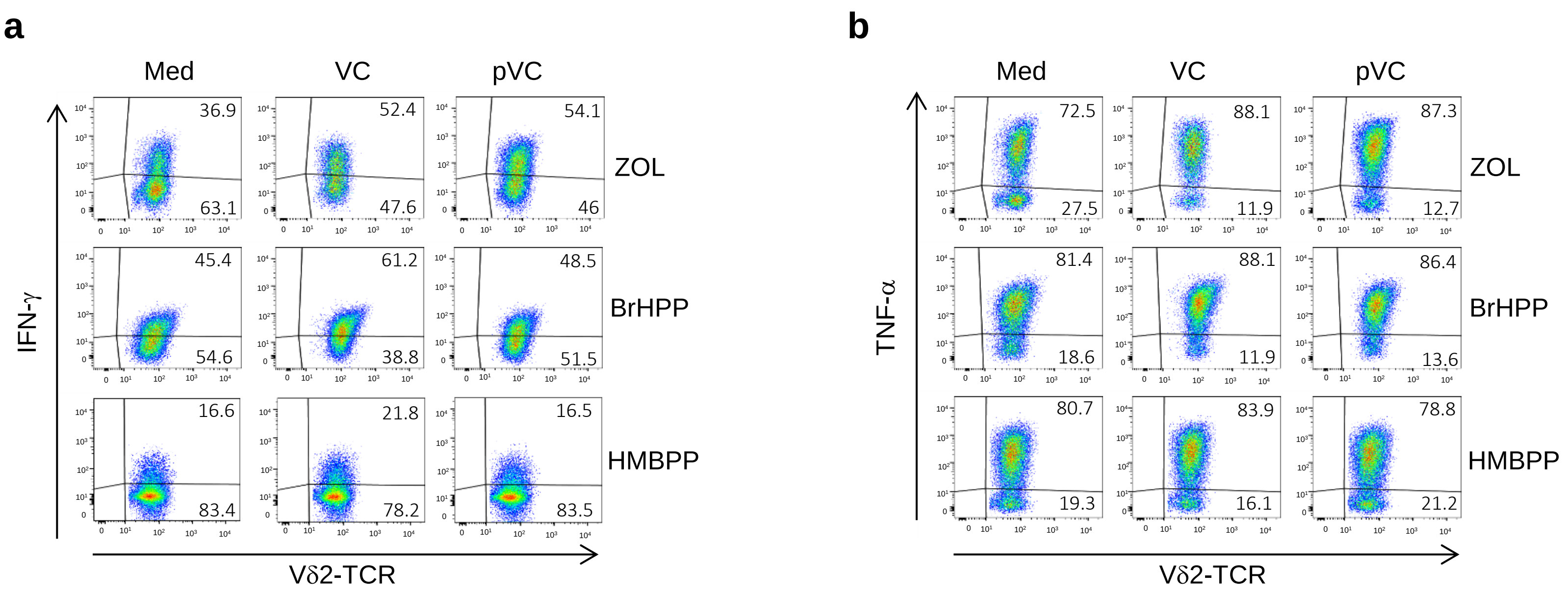

Supplemental Figure S2: Gating strategy to detect intracellular expression of IFN-γ and TNF-α.

PBMC were activated by the indicated γδ T-cell stimuli ZOL, BrHPP or HMBPP in the absence or presence of 12.5 µg/mL (70 µM VC) or 50 µg/mL (173 µM) pVC as indicated. After 14 d, cells were restimulated for 6h with anti-CD3/CD28 mAb in the presence of brefeldin A, and then stained with anti-human CD3-APC-H7, anti-human TCR Vδ2-PercP, before being permeabilized and stained intracellularly with (a) anti-IFN-γ and (b) anti-TNF-α antibodies. For flow cytometric analysis, a gate was set on Vδ2 –positive cells.

Supplemental Figure S3

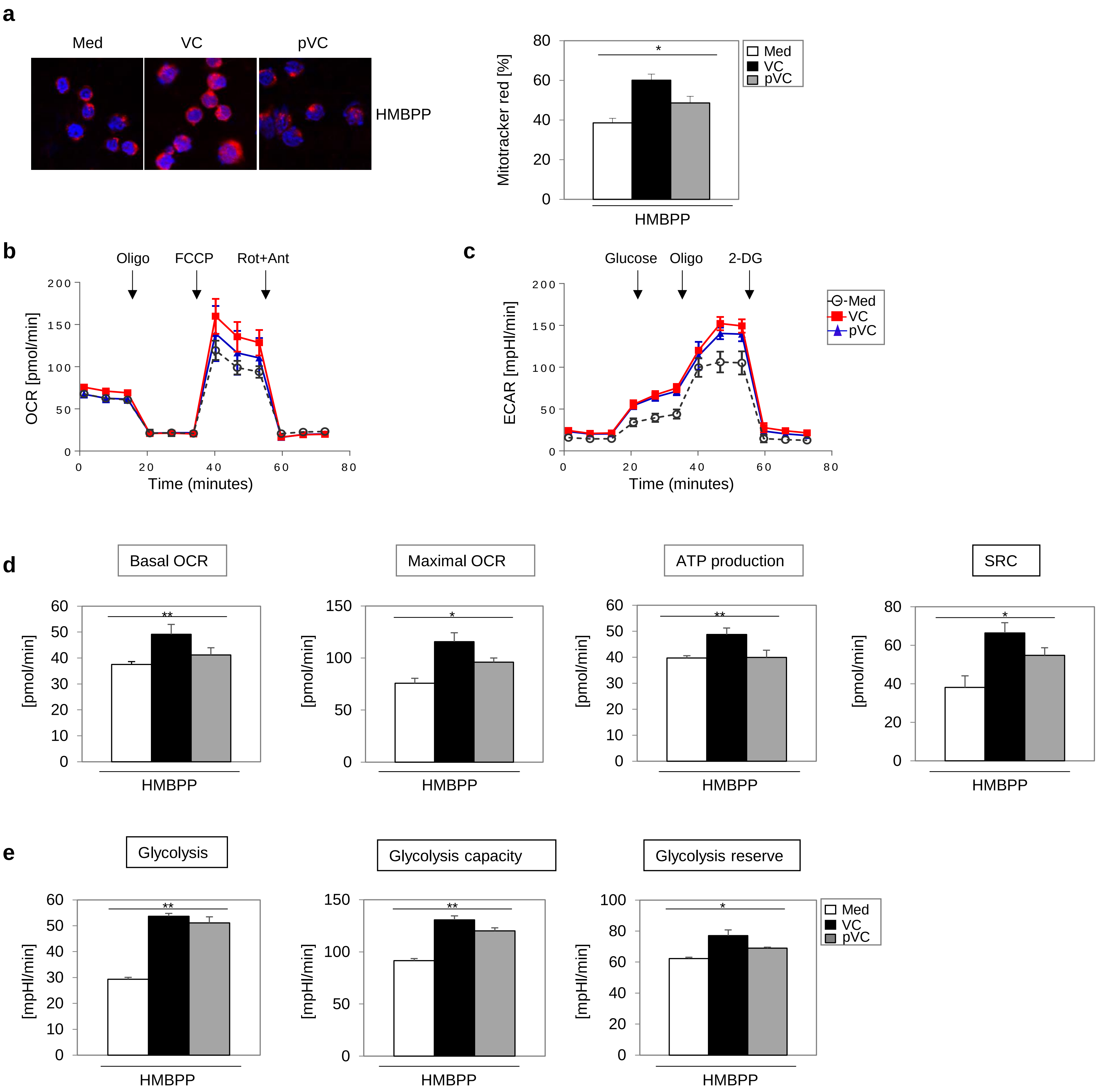

Supplemental Figure S3: Effect of VC and pVC on the metabolic activity of HMBPP-activated γδ T cells. PBMC from 3-4 healthy donors were activated for 14 d with HMBPP in the presence of IL-2 and additional presence or absence of 12.5 µg/mL (70 µM) VC or 50 µg/mL (173 µM) pVC as indicated. **(a)** γδ T cells were stained with TCR Vδ2-PE, MitoTracker™ Red FM, and DAPI. The distribution of mitochondria was observed by confocal microscopy (left) and the fluorescence intensity of mitochondria was measured by flow cytometry (right; mean ± SEM of 3 experiments). **(b, d)** Oxygen Consumption Rates (OCR), ATP production and Spare Respiratory Capacity (SRC), and **(c, e)** glycolysis were measured at d 14 on the XF-96 Extracellular Flux Analyzer (n=3). Statistical significance was calculated with ANOVA. Errors bars represent SEM. * $p<0.05$, ** $p<0.01$

Supplementary Figure S4

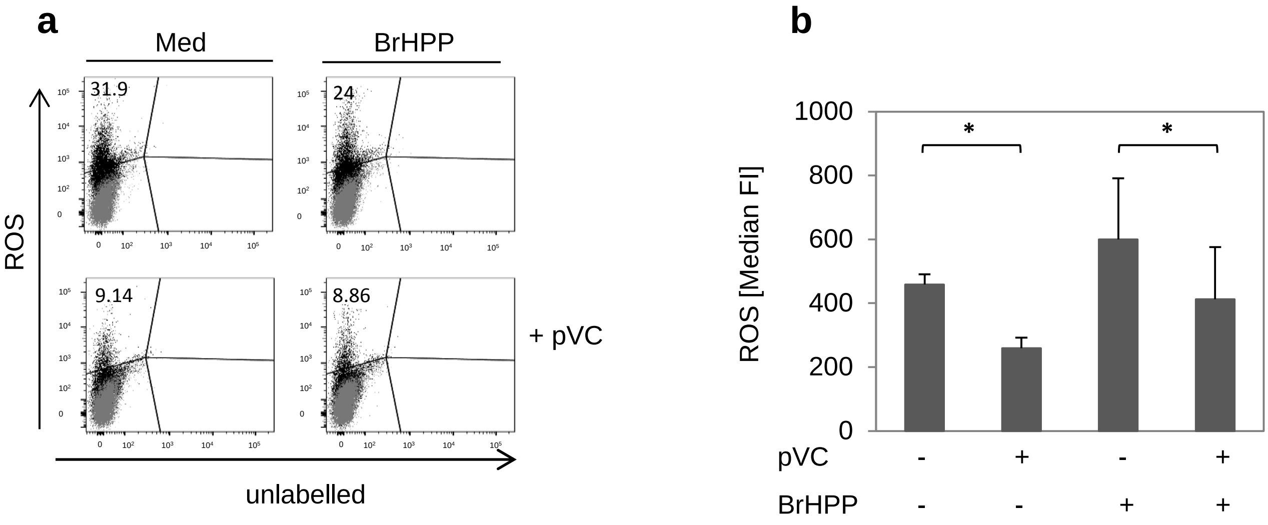

Supplementary Figure S4: pVC decreases the level of intracellular ROS.

12 d short-term expanded Vγ9Vδ2 T-cell lines were left unstimulated or were restimulated with BrHPP for 3h following 1h pretreatment with 50 µg/mL (173 µM) pVC (where indicated). The level of intracellular ROS was measured by flow cytometry using a ROS detection kit. **(a)** Representative dot plots from one experiment are shown. Numbers in the indicated area in FACS plots refer to the percentage of positive cells. **(b)** Summary bar graphs of 4 independent experiments (mean ± SD) showing isotype-subtracted median fluorescence intensity [Median FI]. Statistical significance was calculated with the paired, two-tailed Student's t-test. * $p<0.05$

Supplemental Figure S5

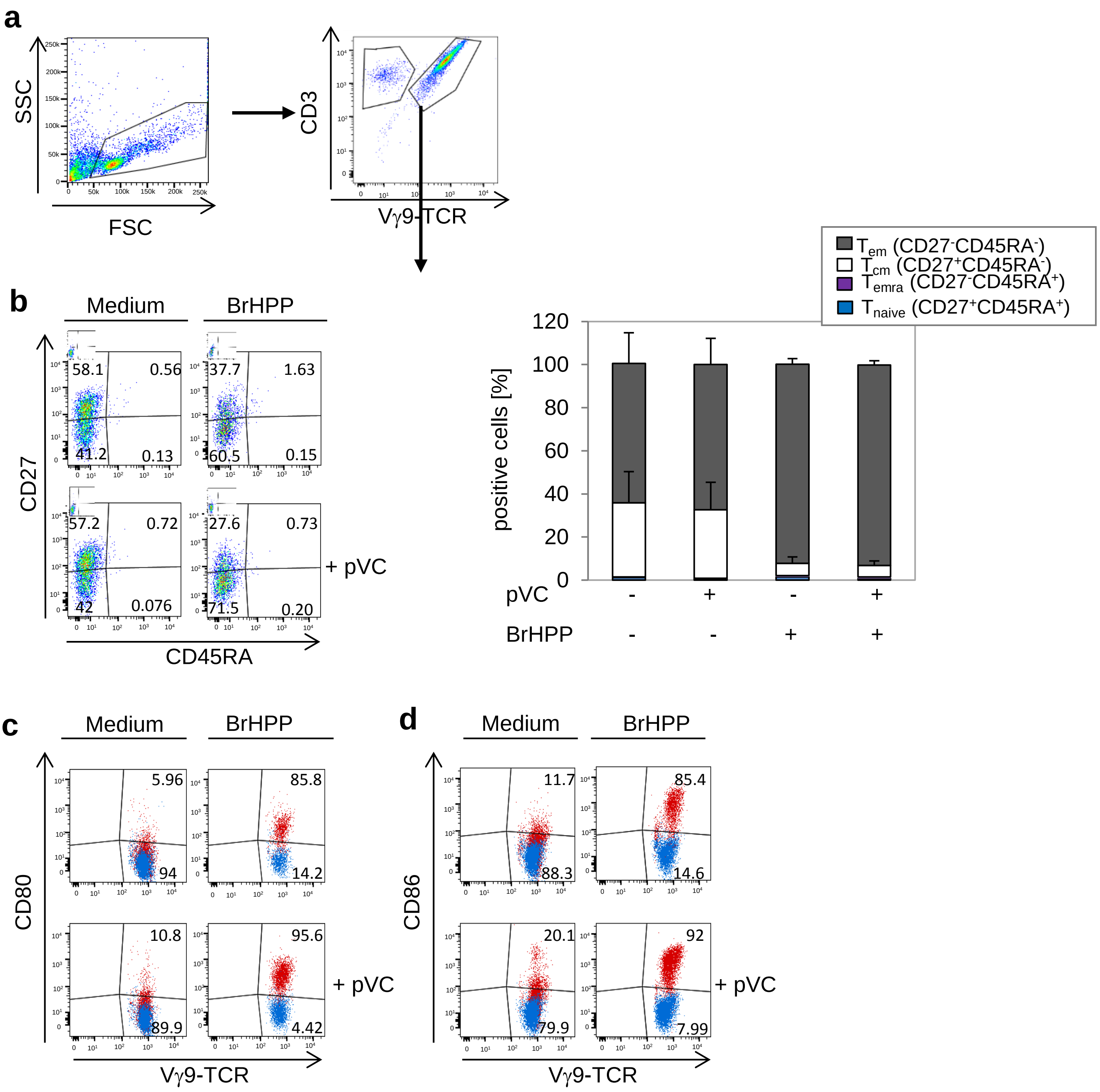

Supplemental Figure S5: Modulation of surface marker expression by pVC.

12 d short-term expanded Vγ9Vδ2 T-cell lines were left unstimulated (Medium) or were restimulated with BrHPP in the absence or presence of 50 µg/mL (173 µM) pVC (where indicated). Surface marker expression was determined on γδ T cells after additional 4 d. **(a)** Gating strategy for analysis of surface markers on Vγ9-positive γδ T cells. **(b)** Expression of CD27 and CD45RA on gated Vγ9 T cells. **Left**: representative dot plot analysis from 1 donor. **Right**: Summary of the distribution of the 4 subsets based on CD27 and CD45RA expression, n=4. **(c) and (d)** Representative dot plots of cell surface expression of **(c)** CD80 (clone L307.4) and **(d)** CD86 (clone 2331[FUN-1])
